# Pressure Losses across Multiple Fittings in Ventilation Ducts

**DOI:** 10.1155/2013/195763

**Published:** 2013-12-09

**Authors:** Z. T. Ai, C. M. Mak

**Affiliations:** Department of Building Services Engineering, The Hong Kong Polytechnic University, Hong Kong

## Abstract

The accurate prediction of pressure losses across in-duct fittings is of significance in relation to the accurate sizing and good energy efficiency of air-delivery systems. Current design guides provide design methods and data for the prediction of pressure losses only for a single and isolated fitting. This study presents an investigation of pressure losses across multiple interactive in-duct fittings in a ventilation duct. A laboratory measurement of pressure losses across one fitting and multiple fittings in a ventilation duct is carried out. The pressure loss across multiple interactive fittings is lower than that across multiple similar individual fittings, while the percentage decrease is dependent on the configuration and combination of the fittings. This implies that the pressure loss across multiple closely mounted fittings calculated by summing the pressure losses across individual fittings, as provided in the ASHRAE handbook and the CIBSE guide, is overpredicted. The numerical prediction of the pressure losses across multiple fittings using the large-eddy simulation (LES) model shows good agreement with the measured data, suggesting that this model is a useful tool in ductwork design and can help to save experimental resources and improve experimental accuracy and reliability.

## 1. Introduction

In air-delivery ductworks of HVAC systems, pressure losses across duct fittings such as dampers, sensors, bends, transition pieces, duct corners, branches, and even splitter attenuators are important in counteracting the pressure difference created by fans. Accurately predicting pressure losses across duct fittings at the design stage is thus crucially important to proper duct sizing and fan selection, which could finally result in great economic benefits in terms of both the initial investment cost and the operational cost of duct systems.

The commonly adopted data of pressure losses across HVAC duct fittings are those provided in the well-known design guides, such as the ASHRAE handbook [1], the CIBSE guide [2], and the handbook by Idelchik [3]. These data were summarized from many experimental works, most of which were conducted based on ASHRAE Standard 120P [4]. However, in terms of scope, the data are limited to the types of duct fittings, the range of duct sizes, and the range of mean duct velocities. In addition, the accuracy of the experimentally obtained data available in these handbooks and guide has been questioned by a number of investigators [5–11]. One possible reason for their inaccuracy is that the measurements were conducted on single, isolated duct fittings without consideration of the influence of the interaction of other fittings [7]. In practice, there are commonly multiple fittings in a HVAC ductwork, and very frequently, some of these are relatively close to each other. Rahmeyer [12] experimentally studied the effect of the interaction between bends and found that the pressure loss across two closely coupled bends is related to their distance. This finding implies that the traditional calculation method, which sums pressure losses across each individual duct bend, could sometimes be inaccurate. Later, Atkin and Shao [7] applied computational fluid dynamics (CFD) modeling to analyze the effects of the separation and orientation of two closely connected bends on total pressure loss. They found that at a separation distance of 8 to 10 hydraulic diameters, the pressure drop across the two bends is highly dependent on their relative orientation. Unfortunately, it is not known whether these findings from bends can be applied to other fittings, especially in-duct fittings. Thus, more studies are required.

Another concern is that obvious differences are found in part of the pressure loss data between the ASHRAE handbook and the CIBSE guide [6]. One factor that may possibly contribute to these discrepancies [6] is that due to the lack of understanding on airflow patterns at duct fittings, pressure sensors are occasionally located at inappropriate sections such as disturbance sections, which could cause large measurement errors. However, since the in-duct airflow pattern is strongly associated with the duct configuration, mean duct velocity, and local aerodynamic configuration of fittings, it is difficult and time consuming to find the appropriate locations for placing pressure sensors before every test. In such a condition, a numerical method should be helpful in terms of saving experimental investment and time. Even in an experimental way, it is more reliable and economical to know the flow patterns before a real test is set up and conducted. As a numerical method, CFD has been sufficiently verified and validated as a method of predicting fluid flow. Shao and Riffat [6, 8] studied the possibility and accuracy of using the CFD method to predict the pressure loss factor and in turn determine pressure losses across duct bends. They evaluated the effect of a set of computational parameters on the accuracy of numerical results. Their numerical results are supported by the experiments of Gan and Riffat [13]. In addition, the CFD method has been used to predict the pressure loss coefficient of many other duct fittings, such as damper, orifice, transition [9–11, 14], and junction [15] fittings, in HVAC ductworks as well as to conduct other duct-related studies (e.g., air leakage) [16]. Without exception, these previous numerical simulations used the Reynolds Averaged Navier-Stokes (RANS) method [17–21], specifically the steady standard *k*-*ε* turbulence model. However, this model may be not accurate and reliable enough to predict the flow field inside a duct with multiple fittings, where the airflow is more strained and swirling as well as highly fluctuating. As an alternative CFD model, the advanced large-eddy simulation (LES) model is well known for its accuracy in predicting airflow in the building-related field [22, 23]. The LES model, which resolves large turbulent eddies and models small eddies, has the capacity to reproduce transient turbulent fluctuations and handle flow intermittency, although it consumes more numerical cost. In this study, the accuracy and reliability of the LES turbulence model in predicting pressure losses across multiple in-duct fittings are evaluated.

The specific problems that motivated this study are the inaccuracy of the available data in the current guides and the lack of a predictive method for pressure losses across multiple in-duct fittings. The purposes of this study, therefore, are to examine the pressure losses across multiple in-duct fittings and to evaluate the accuracy and reliability of a predictive method. The effect of the interaction of fittings on the total pressure loss across multiple fittings is analyzed by experimental tests. The predictive method, namely, LES modeling, is then evaluated by comparing it with tested data. It is expected that this study will reveal the pressure losses across multiple in-duct fittings and provide designers with a predictive method that can be used either independently as a design tool or to assist experimental testing.

## 2. Conceptual Models

There are two types of pressure loss in duct systems, namely, friction loss and dynamic loss. These losses are derived from different mechanisms and are therefore calculated by different methods [1].

Friction loss is due to fluid viscosity and results from the momentum exchange between molecules or between adjacent fluid layers moving at different velocities. It occurs along the entire length of a duct. Friction losses in fluid ductworks can be calculated by the Darcy equation:

(1) $$\begin{array}{c} \Delta P_{f}=\frac{fL}{D_{h}}\times \frac{\rho U^{2}}{2}, \end{array}$$

where Δ*P*_*f*_ is a friction loss, *f* a dimensionless friction factor, *L* duct length, *D*_*h*_ hydraulic diameter, *U* area-averaged streamwise velocity, and *ρ* fluid density. Friction factor *f* is determined by the Colebrook equation:

(2) 1f=−2log⁡(e3.7Dh+2.51Ref),

where *e* is the absolute roughness factor of a material and *Re* is the Reynolds number computed from

(3) Re=DhUν,

where *ν* is kinematic viscosity. The hydraulic diameter *D*_*h*_ is defined as 4*A*/*P*, where *A* is the duct area and *P* is the perimeter of the cross-section.

Dynamic losses in fittings result from flow disturbances caused by duct fittings that change the airflow direction or flow path area and can be calculated by

(4) $$\begin{array}{c} \Delta P_{d}=C\times \frac{\rho U^{2}}{2}, \end{array}$$

where *C* is the dimensionless local loss coefficient (also called *k* factor), which is determined by the local dynamic characteristics.

## 3. Experimental Method and Simplification

**Figure 1:**
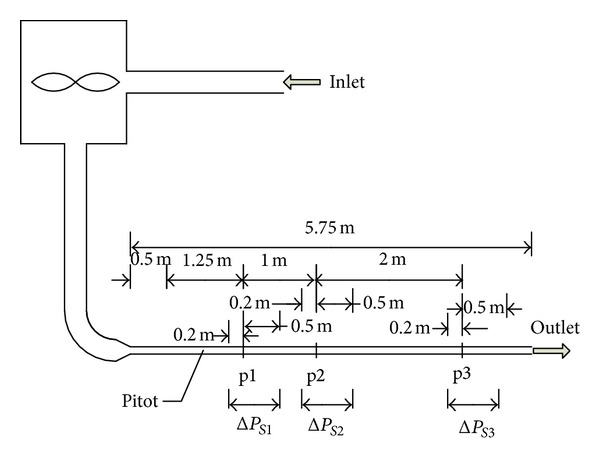
A schematic diagram of the experimental system (p1, p2, and p3 are the positions where the first, second, and third fittings are inserted, respectively; Δ*P*_*S*1_, Δ*P*_*S*2_, and Δ*P*_*S*3_ represent the static pressure loss across the first, second, and third fittings, resp.).

This experiment was a part of our previous test on flow noise caused by in-duct elements [24]. The experimental system is shown in Figure 1. Air flow was provided by a centrifugal fan driven by a variable speed motor. The fan was enclosed in a 1.22 × 1.22 × 1.22 m^3^ enclosure. The 0.1 × 0.1 m^2^ test duct was made of steel. The total length of the duct was 5.75 m, in which, counted from the air flow inlet, the first fitting (p1) was located 1.75 m from the duct inlet section and the third fitting (p3) was located 1 m from the duct outlet. These upstream and downstream lengths [6] were generally sufficient to ensure that the test of the first and third fittings were not affected by the inlet and outlet of the duct, respectively. The inlet and outlet of the experimental system were placed on the outside so as to eliminate the effect of relative pressure difference.

**Figure 2:**
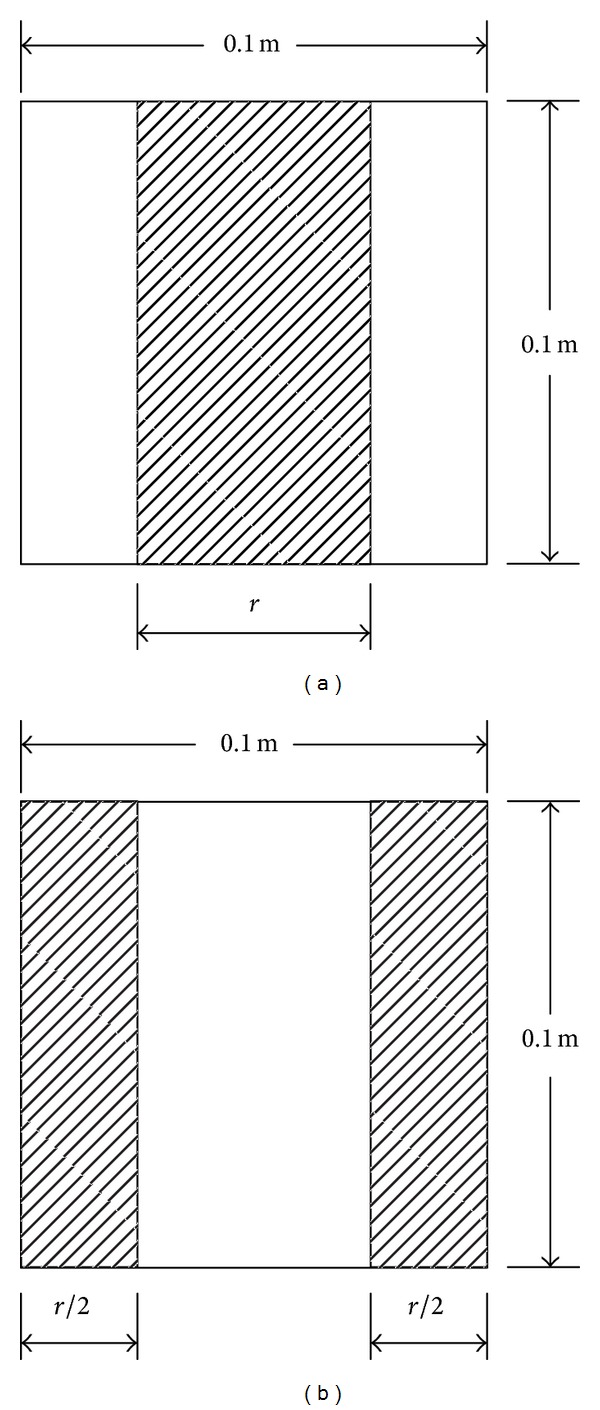
Cross-section of the duct with two types of flat-plate fittings (shaded area). (a) Centrally placed fittings; *r* = 0.025 m, 0.05 m, 0.075 m. (b) The geometries consisted of plates protruding symmetrically from both sides of the duct, leaving a central vertical strip of the duct open, later called a centrally opened fitting; *r* = 0.025 m, 0.05 m, 0.075 m.

As shown in Figure 2, flat plates were used to generally represent the in-duct fittings in HVAC ductworks. Here, the control variable was the obstructed ratio of the fitting area to the cross-sectional area of the duct. The plates were made from 1 mm thick steel plate, and they were fixed to the adjoining flanges of the test duct. The gap was sealed with compressed foam rubber. As shown in Table 1, 15 configurations were tested. In Cases 1–6, only a single fitting was inserted into position p1. In Cases 7–13, two fittings were inserted at two different positions (p1 and p2), and in Cases 14-15, three fittings were inserted at three different positions (p1, p2, and p3).

The velocity profile in the empty test duct was measured to make sure that the flow could be symmetrically developed inside the duct. A pitot tube was used to sample the dynamic pressure at specified points in the duct cross-section. Based on the measurements obtained using this empty duct, a relationship between the mean duct velocity (*y*) and the velocity measured at the center of the duct (*x*) was developed (*y* = 0.9639*x* − 0.3289 (*R* ^2^ = 0.9997)), and this was used to calibrate the mean duct velocity in later tests using the measured center velocity. The mean airflow velocities tested for each case are listed in Table 1.

The static pressure losses across the fittings were measured using two piezometric rings placed at positions p1, p2, and p3 (Figure 1). Each ring consisted of four static pressure tappings, one in each duct face. The downstream ring was sufficiently far away (five times the duct dimensions) from the test fitting to ensure that full static pressure recovery could take place in the wake of the flow obstructions under test.

## 4. Numerical Modeling

This section briefly discusses the numerical method used by LES modeling and introduces the test cases selected to evaluate it.

### 4.1. Governing Equations of LES

The governing equations used for large eddies can be obtained by filtering the time-dependent Navier-Stokes equations. Eddies whose scales are smaller than the filtering width or grid spacing adopted in the computations are effectively removed by the filtering process. Then, the resulting equations only govern the dynamics of large eddies.

In this study, the filtering operation provided by the finite volume discretization method, as described in [25], is employed:

(5) $$\begin{array}{c} \bar{\varphi }\left(x\right)=\frac{1}{V}\int_{0}^{V}\varphi \left(x^{\prime }\right)dx^{\prime },\;x^{\prime }\in V, \end{array}$$

where $\bar{\varphi }\;\left(x\right)$ represents a filtered variable and *V* the volume of a computational control cell. The filter function, *G*(*x*, *x*′), is

(6) $$\begin{array}{c} G\left(x,x^{\prime }\right)=\frac{1}{V},\;x^{\prime }\in V\;\text{or}\;G\left(x,x^{\prime }\right)=0,\;x^{\prime }\notin V. \end{array}$$

In this study, the governing equations of LES for incompressible flows were obtained by filtering the Navier-Stokes equations:

(7) $$\begin{array}{c} \frac{\partial \bar{u_{i}}}{\partial x_{i}}=0,\\ \frac{\partial }{\partial t}\left(\rho \bar{u_{i}}\right)+\frac{\partial }{\partial x_{j}}\left(\rho \bar{u_{i}}\;\bar{u_{j}}\right)=\frac{\partial }{\partial x_{j}}\left(\sigma_{ij}\right)-\frac{\partial \bar{p}}{\partial x_{i}}-\frac{\partial \tau_{ij}}{\partial x_{j}}, \end{array}$$

where *σ*_*ij*_ is the stress tensor due to molecular viscosity, defined by

(8) $$\begin{array}{c} \sigma_{ij}\equiv \left[\mu \left(\frac{\partial \bar{u_{i}}}{\partial x_{j}}+\frac{\partial \bar{u_{j}}}{\partial x_{i}}\right)\right]-\frac{2}{3}\mu \frac{\partial \bar{u_{i}}}{\partial x_{i}}\delta_{ij} \end{array}$$

and *τ*_*ij*_ is the subgrid-scale (SS) stress defined by

(9) $$\begin{array}{c} \tau_{ij}\equiv \rho \bar{u_{i}u_{j}}-\rho \bar{u_{i}}\bar{u_{j}}. \end{array}$$

Since the subgrid-scale stress term in the LES model is unknown, it requires modeling to close the governing equations. Currently, the most adopted subgrid-scale turbulence model, which employs the Boussinesq hypothesis [26], computes subgrid-scale turbulent stresses by

(10) $$\begin{array}{c} \tau_{ij}-\frac{1}{3}\tau_{kk}\delta_{ij}=-2\mu_{t}\bar{S_{ij}}, \end{array}$$

where *μ*_*t*_ is subgrid-scale turbulent viscosity. The isotropic part, *τ*_*kk*_, which is not modeled, is added to the filtered static pressure term. *S*_*ij*_ is the rate-of-strain tensor under the resolved scale, defined by

(11) $$\begin{array}{c} S_{ij}=\frac{1}{2}\left(\frac{\partial \bar{u_{i}}}{\partial x_{j}}+\frac{\partial \bar{u_{j}}}{\partial x_{i}}\right). \end{array}$$

In this study, the subgrid-scale turbulent viscosity *μ*_*t*_ was modeled by the Smagorinsky-Lilly model, which was initially proposed by Smagorinsky [27]. In the Smagorinsky-Lilly model, the turbulent viscosity coefficient is calculated by

(12) $$\begin{array}{c} \mu_{t}=\rho L_{S}^{2}\left|\bar{S}\right|, \end{array}$$

where $\left|\bar{S}\right|\equiv \sqrt{2\bar{S_{ij}S_{ij}}}$, *L*_*S*_ is the subgrid mixing length, calculated by

(13) $$\begin{array}{c} L_{S}=\min\left(\kappa d,C_{S}V^{1/3}\right), \end{array}$$

where *κ* is the von Kaman constant, *d* the distance to the closest wall, and *C*_*S*_ the Smagorinsky constant, empirically given as 0.1.

### 4.2. Mesh Work, Boundary Conditions, and Numerical Scheme

**Figure 3:**
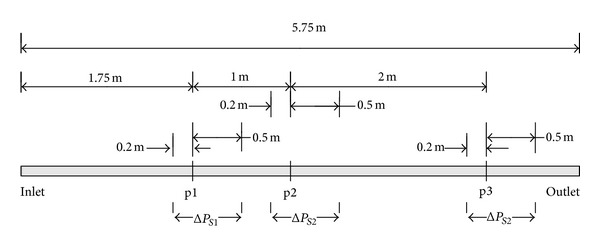
A schematic diagram of the duct system in the numerical simulations.

In this study, a full straight square duct (0.1 × 0.1 m^2^ in section and 5.75 m in length) was simulated (see Figure 3). The fluid air was assumed to be incompressible, and the gravitational acceleration was not considered. The Reynolds number based on the mean duct velocity and square duct dimensions was 0.67–1.47 × 10^5^. Mean duct velocity was imposed on the inlet boundary and the flow turbulence is characterized by turbulence intensity (10%) and hydraulic diameter (0.1 m). On the outlet boundary, it is assumed that the flow is fully developed, with zero normal gradients and zero background pressure. There was no slip of fluid at the surfaces of the duct and fitting(s). Structured grids were used to discretize the computational domain in which the first grids are 2.5 × 10^−5^ m away from the fitting(s). Then, the *y* ^+^ (*y* ^+^ = *ρu*_*τ*_ *y*_*p*_/*μ*) value for the first grid points was around 0.5–2 depending on the mean duct velocity, which indicates that the first grids are within the laminar sublayer. The meshes become coarser in the region far away from the fitting(s) but remain high density near the duct walls (*y* ^+^ < 5). When the mesh is fine enough to resolve the laminar sublayer, the LES model applies the laminar stress-strain relationship to obtain the wall shear stress:

(14) $$\begin{array}{c} \frac{\bar{u}}{u_{\tau }}=\frac{\rho u_{\tau }y}{\mu }. \end{array}$$

The sensitivity of mesh number was systematically tested. For each case, three different mesh systems (a coarser, a medium, and a finer) were constructed and the final numerical solutions based on these three meshes were compared. Finally, in compromise between the numerical accuracy and cost, meshes with around 2.0 × 10^6^, 2.5 × 10^6^, and 3.0 × 10^6^ grids were selected for the cases with one fitting, two fittings, and three fittings, respectively. The time step size used in the LES simulations was 0.0002 s, which ensures that the convergence can be achieved within 5–10 iterative steps for each time step.

Based on the finite volume method (FVM), the governing equations are discretized to algebraic equations on the grid system. The convection term was discretized by the bounded central differencing scheme, while the pressure staggering option scheme (PRESTO) was selected for pressure interpolation. Finally, the SIMPLEC algorithm was used to couple the pressure and velocity equations.

### 4.3. Cases Simulated

In order to evaluate the accuracy and reliability of the LES model in predicting the pressure losses across multiple in-duct fittings, two tested cases are selected to be numerically reproduced: Case 7 at a mean flow velocity of 20 m*/s* and Case 14 at 19 m*/s*. The predicted pressure losses are compared with those measured in the experiments.

## 5. Results and Discussions

As shown in Table 1, five mean flow velocities were tested for each case. However, due to the difficulty in accurately controlling the mean velocity during the tests, the tested velocities were not necessarily the same for all cases. This does not influence the later analysis. In this section, the measured or simulated pressure losses (Pa) across in-duct fittings are directly presented and analyzed. If one is interested in the *k* factors, these can be obtained using (5) in Section 2.

### 5.1. Effect of Reynolds Number (*Re*)

**Figure 4:**
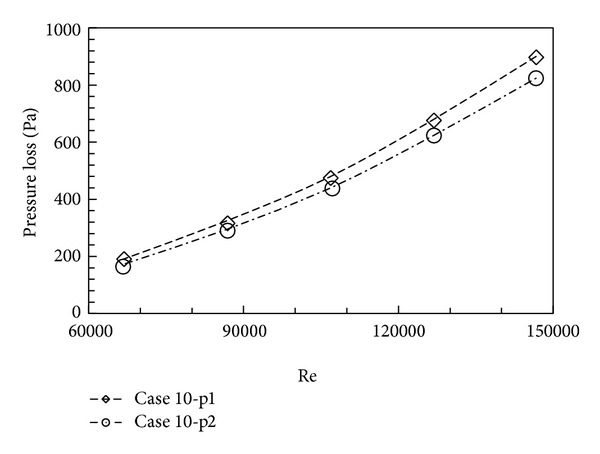
Effect of Reynolds number on pressure loss across fitting(s).

In practice, there are various types of HVAC ducts in terms of cross-sectional shape and dimension and mean flow velocity. Despite this complexity, the dimensionless *Re* can be used to represent these duct characteristics given that the same *Re* indicates aerodynamic similarity. In this section, the effect of *Re* on the pressure losses across fittings is examined when the fitting configuration remains unchanged. It is found that the pressure loss across a fitting almost has a linear relationship with the duct *Re* (the example of Case 10 is shown in Figure 4). This implies that any factors increasing the duct *Re*, such as an increase in velocity and cross-sectional dimensions, can result in an increase in pressure loss across an in-duct fitting. In other words, pressure losses across the in-duct fitting(s) of a larger duct with a higher velocity remain high.

### 5.2. Effect of Fitting Configuration

In order to study the effect of fitting configuration on the pressure losses, this section discusses the cases with the same *Re* to exclude the influence of *Re* on the comparison of different fitting configurations.

The effect of fitting type on pressure losses across fittings is studied when the obstruction ratio is kept constant. The obstruction ratio is defined as the area ratio of the fitting to the duct cross-section, namely, the ratio of the shaded area to the whole duct section (see Figure 2).  Table 2 summarizes the comparison of pressure losses across the two types of fittings, namely, the centrally placed fitting and the centrally opened fitting (in Figure 2). From Table 2, it can be seen that the pressure loss across a centrally placed fitting is remarkably larger than that across a centrally opened fitting. This can be explained by the fact that the velocity profile in the cross-section of a duct follows a parabolic distribution, namely, the largest in the center and the smallest on the duct surfaces. Thus, centrally placed fittings obstruct the fastest central airflow and lead to the largest pressure losses, whereas centrally opened fittings allow this strongest airflow to pass through and offer much less resistance to the airflow. It can also be observed that the deviation ratio of pressure loss between these two types of fittings is not the same and is dependent on the obstruction ratio.

**Figure 5:**
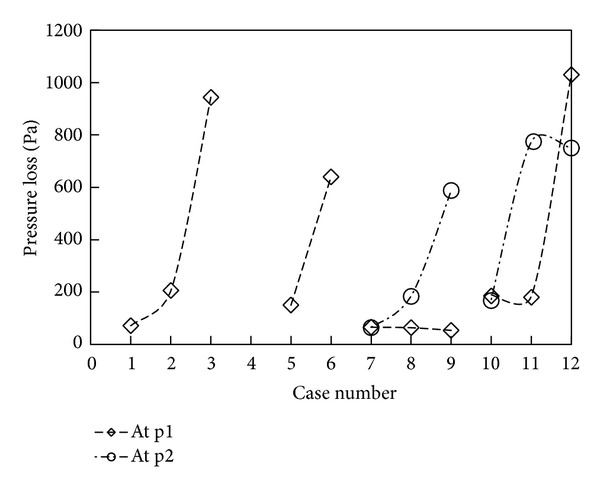
Effect of obstruction ratio on pressure losses across fitting(s).

The effect of the obstruction ratio on pressure losses across fittings is studied at a *Re* of 6.7 × 10^4^, and the results are shown in Figure 5. For all cases, an increase in the fitting's obstruction ratio significantly increases the pressure loss across it. However, the percentage increase in pressure loss is dependent on the obstruction ratio, fitting type, and the interaction of neighboring fittings. For a centrally placed single fitting (Cases 1–3), when the obstruction ratio increases from 0.5 to 0.75 (from Case 2 to 3), the percentage increase is approximately 363%, which is almost one time higher than the increase (183%) when the obstruction ratio increases from 0.25 to 0.5 (from Case 1 to 2). It is also observed that this percentage increase is relatively lower in the case with the centrally opened fitting (Cases 5-6) and is also influenced by the presence of neighboring fittings (Cases 7–9 and Cases 10–12). However, regardless of the change in the obstruction ratio of a neighboring fitting, the pressure loss across a fitting is only changed slightly.

### 5.3. Effect of Interaction of Multiple Fittings

Table 3 summarizes the pressure loss across an upstream fitting and its percentage decrease as a result of its downstream fittings. From Table 3(a), it can be seen that the presence of a downstream centrally placed fitting can reduce the pressure loss across its upstream fitting, and the percentage decrease increases remarkably with the increase in the obstruction ratio of the downstream fitting. However, a comparison of Table 3(a)–(c) shows that with the increase in the obstruction ratio of the upstream fitting, the decrease in pressure loss across it is gradually decreased, becoming −9.1% when the obstruction ratio reaches 0.75. As tabulated in Table 3(d), for the centrally opened fitting, the presence of a downstream fitting can reduce the pressure loss across it, whereas two downstream fittings complicate this situation.

The effect of upstream fittings on the pressure loss across a downstream fitting is also evaluated, and the results are presented in Table 4. For the centrally placed fitting, the presence of upstream fittings significantly reduces the pressure losses across its downstream fitting(s) (see Table 4(a)–(c)). Contrarily, for the centrally opened fitting, this pressure loss is negligibly affected by the presence of an upstream fitting, whereas it is significantly decreased by the presence of two upstream fittings (see Table 4(d)).

The above results suggest that as a result of the effect of downstream and upstream fittings, the pressure loss across an in-duct fitting is changed substantially. This can be explained by the fact that the presence of a fitting changes the airflow direction and turbulence around its neighboring fitting(s) and consequently modifies the *k* factor (see (5) in Section 2). The results also demonstrate that the effect of a neighboring fitting is complex; that is, different fitting type, location (upstream or downstream), obstruction ratio, and duct *Re* can result in very distinctive pressure losses. This implies that the *k* factors for individual fittings provided in the ASHRAE handbook and the CIBSE guide are inaccurate in a condition when the interaction of neighboring fittings cannot be ignored.

The pressure losses across multiple interactive and individual in-duct fittings are compared (see Table 5). In Table 5, the pressure losses across interactive fittings for Cases 7–15 are directly measured in the tests. In order to evaluate the effect of the fittings' interaction on the total pressure loss, for each case, the pressure losses across every individual fitting are summed for comparison. Taking Case 8 as an example, the pressure losses across its individual fittings are summed from Case 1 and Case 2. Based on the summations of individual pressure loss, the percentage decreases in pressure losses across multiple fittings are calculated. It can be seen that the pressure losses across multiple interactive fittings are lower than those across multiple individual fittings, and the percentage decrease is dependent on the configuration and combination of the fittings. This finding is supported by the previous study on two bends by Rahmeyer [12]. Again, this demonstrates that the calculation of pressure losses across multiple closely mounted fittings via summing those across individual fittings is inaccurate. This method overpredicts the total pressure loss, which may consequently result in energy waste owing to the selection of larger fans. In such a condition, exploring an accurate, reliable, and high-efficiency predictive method, such as a validated CFD model, is crucially important.

### 5.4. Validation of LES Modeling

In order to validate the LES model in predicting pressure losses across multiple in-duct fittings, the predicted values of the pressure losses across fittings in Case 7 at 20 m/s and Case 14 at 19 m/s are compared with corresponding data measured in the tests. The results are presented in Table 6. It can be seen that the predicted results agree well with the measured data, with a relative deviation less than 3%. This indicates that the LES model can accurately resolve the flow field in a HVAC duct with multiple in-duct fittings.

**Figure 6:**
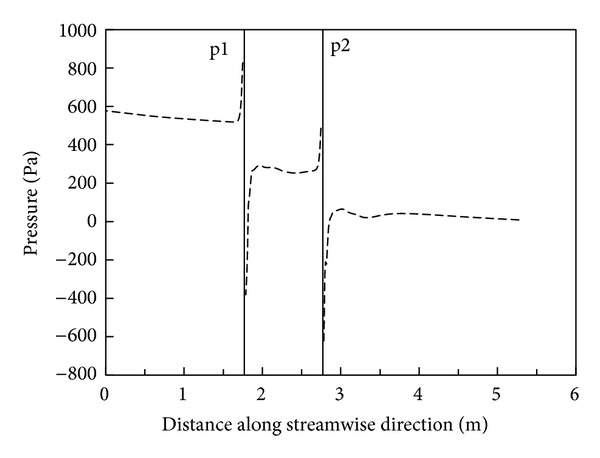
Pressure distribution along centerline of the duct (Case 7 at 20 m/s).

**Figure 7:**
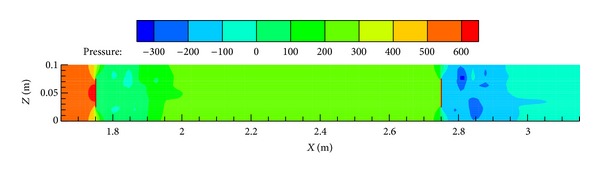
Pressure (Pa) contour around the in-duct fittings on the center plane of the duct (Case 7 at 20 m/s).

**Figure 8:**
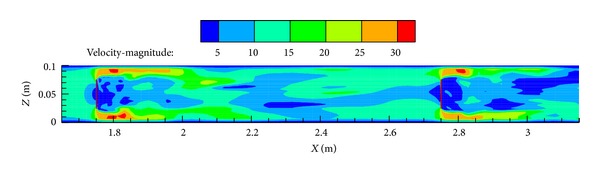
Air speed (m/s) contour around the in-duct fittings on the center plane of the duct (Case 7 at 20 m/s).

Compared to the experimental measurement, numerical modeling has an incomparable advantage in obtaining in-duct flow details such as velocity and pressure distributions.  Figure 6 presents the pressure distribution along the duct centerline in Case 7 at a mean flow velocity of 20 m/s. Figures 7 and 8 show the pressure and air speed contours on the center plane of the duct, respectively. These kinds of pressure and air speed distribution are useful because it can not only be used independently for ductwork design (if the numerical model is validated before) but also to indicate the locations where pressure sensors should be placed in the tests; for the latter, the involvement of numerical modeling can save many experimental resources and help to produce more reliable experimental data. Thus, the successful use of numerical modeling is of great significance in optimizing ductwork design and improving the database for pressure losses across fittings.

## 6. Conclusions

This study examines the pressure losses across multiple in-duct fittings of a ventilation duct using experimental tests. Two tested cases are reproduced by LES modeling to evaluate the accuracy and reliability of this numerical method in predicting the pressure field inside a duct with multiple fittings. The following conclusions can be drawn.

The flow resistance of a centrally placed fitting is remarkably larger than that of a centrally opened fitting basically due to the fact that the velocity profile of a cross-section of a duct follows a parabolic distribution. For all cases, an increase in the obstruction ratio of a fitting significantly increases the pressure loss across it. However, this pressure loss does not linearly increase with the obstruction ratio; there is a substantial increase in pressure loss when the obstruction ratio increases from 0.5 to 0.75. Again, this is because the velocity profile on a cross-section is not a uniform distribution.

Since the presence of a fitting changes the airflow direction and turbulence around its close neighboring fitting(s) and consequently modifies the *k* factor, the pressure losses across the neighboring fitting(s) are changed substantially. However, the magnitude of this change is affected by many factors, such as fitting type, location (upstream or downstream), obstruction ratio, and *Re*. In addition, the pressure losses across multiple interactive fittings are lower than those across multiple similar individual fittings, although the percentage decrease is dependent on the configuration and combination of the fittings. These findings imply that the calculation of pressure losses across multiple closely mounted fittings via summing those across individual fittings is inaccurate. This method overpredicts the total pressure loss and could result in energy waste via the selection of larger fans. Thus, a more accurate, reliable, and high-efficiency predictive method, such as a validated CFD model, should be explored.

The predicted results by LES modeling agree well with the measured data in the tests, which demonstrates that the LES model can accurately resolve the flow field in a HVAC duct with multiple in-duct fittings. Compared to the experimental measurement, the numerical modeling can provide the details of pressure distribution. This predicted pressure distribution can be used not only independently in a ductwork design (if the numerical model has been validated before) but also to assist in tests to find correct locations to place pressure sensors. In the latter case, the use of numerical modeling can potentially save many experimental resources and help to produce more reliable experimental data.

## Figures and Tables

**Table 1:** Fifteen fitting configurations tested in this study.

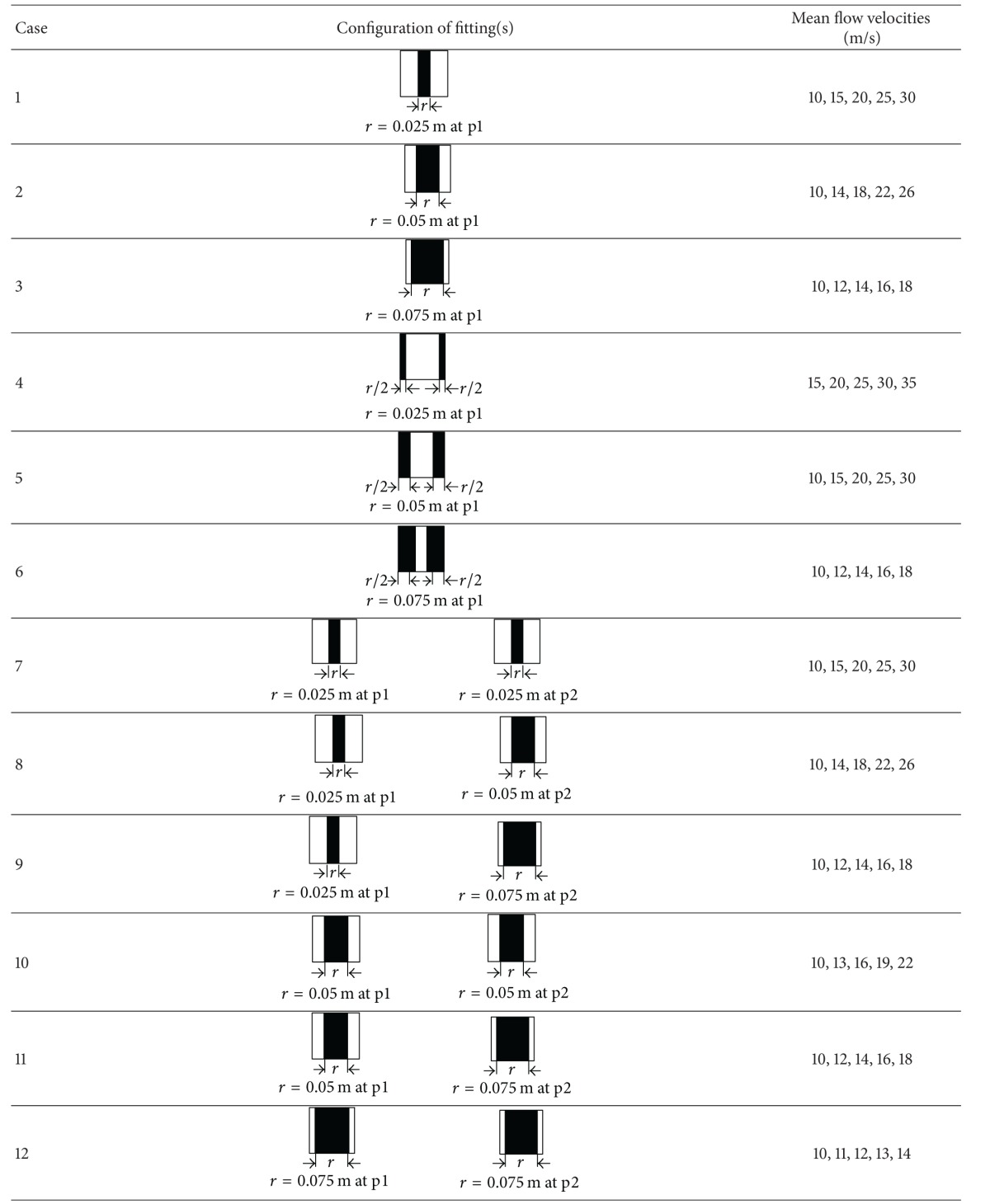 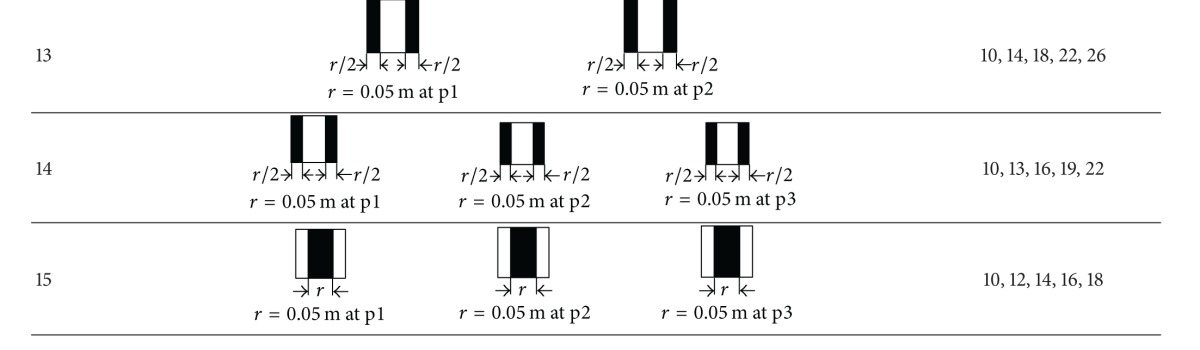

**Table 2:** Effect of fitting type on pressure losses (Pa) across fitting(s).

Re (×10^4^)	Case 1 versus 4 (only p1)	Case 2 versus 5 (only p1)	Case 3 versus 6 (only p1)	Case 10 versus 13 (p1 and p2)	Case 15 versus 14 (p1, p2 and p3)
6.7	—	204 versus 150	944 versus 640	190 versus 144 at p1 172 versus 150 at p2	208 versus 142 at p1 198 versus 154 at p2 180 versus 118 at p3
13.3	286 versus 144	—	—	—	—

**Table:** (a) Base case: Case 1

Re = 6.67 × 10^4^	Case 1	Case 7		Case 8		Case 9	
	Δ*P*_*S*1_	Δ*P*_*S*1_	Δ*P*_*S*2_	Δ*P*_*S*1_	Δ*P*_*S*2_	Δ*P*_*S*1_	Δ*P*_*S*2_
Pressure loss	72	66	66	64	185	54	588
Percentage decrease		8.3%		11.1%		25.0%	

**Table:** (b) Base case: Case 2

Re = 6.67 × 10^4^	Case 2	Case 10		Case 11		Case 15		
	Δ*P*_*S*1_	Δ*P*_*S*1_	Δ*P*_*S*2_	Δ*P*_*S*1_	Δ*P*_*S*2_	Δ*P*_*S*1_	Δ*P*_*S*2_	Δ*P*_*S*3_
Pressure loss	204	190	172	184	774	208	198	180
Percentage decrease		6.9%		9.8%		−2.0%	2.9%	

**Table:** (c) Base case: Case 3

Re = 6.67 × 10^4^	Case 3	Case 12	
	Δ*P*_*S*1_	Δ*P*_*S*1_	Δ*P*_*S*2_
Pressure loss	944	1030	750
Percentage decrease		−9.1%	

**Table:** (d) Base case: Case 5

Re = 6.67 × 10^4^	Case 5	Case 13		Case 14		
	Δ*P*_*S*1_	Δ*P*_*S*1_	Δ*P*_*S*2_	Δ*P*_*S*1_	Δ*P*_*S*2_	Δ*P*_*S*3_
Pressure loss	150	144	150	142	154	118
Percentage decrease		4.0%		5.3%	−2.7%	

**Table:** (a) Base case: Case 1

Re = 6.67 × 10^4^	Case 1	Case 7	
	Δ*P*_*S*1_	Δ*P*_*S*1_	Δ*P*_*S*2_
Pressure loss	72	66	66
Percentage decrease			8.3%

**Table:** (b) Base case: Case 2

Re = 6.67 × 10^4^	Case 2	Case 8		Case 10		Case 15		
	Δ*P*_*S*1_	Δ*P*_*S*1_	Δ*P*_*S*2_	Δ*P*_*S*1_	Δ*P*_*S*2_	Δ*P*_*S*1_	Δ*P*_*S*2_	Δ*P*_*S*3_
Pressure loss	204	64	185	190	172	208	198	180
Percentage decrease			9.3%		15.7%		2.9%	11.8%

**Table:** (c) Base case: Case 3

Re = 6.67 × 10^4^	Case 3	Case 9		Case 11		Case 12	
	Δ*P*_*S*1_	Δ*P*_*S*1_	Δ*P*_*S*2_	Δ*P*_*S*1_	Δ*P*_*S*2_	Δ*P*_*S*1_	Δ*P*_*S*2_
Pressure loss	944	54	588	184	774	1030	750
Percentage decrease			37.7%		18.0%		20.6%

**Table:** (d) Base case: Case 5

Re = 6.67 × 10^4^	Case 5	Case 13		Case 14		
	Δ*P*_*S*1_	Δ*P*_*S*1_	Δ*P*_*S*2_	Δ*P*_*S*1_	Δ*P*_*S*2_	Δ*P*_*S*3_
Pressure loss	150	144	150	142	154	118
Percentage decrease			0.0%		−2.7%	21.3%

**Table 5:** Comparison of pressure losses (Pa) across multiple interactive and individual fittings.

Re = 6.67 × 10^4^	Case 7	Case 8	Case 9	Case 10	Case 11	Case 12	Case 13	Case 14	Case 15
Across interactive fittings	132	249	642	362	958	1780	294	414	586
Across individual fittings	144	276	1016	408	1148	1888	300	450	612
Percentage decrease	8.3%	9.8%	36.8%	11.3%	16.6%	5.7%	2.0%	8.0%	4.2%

**Table 6:** Comparison of predicted and measured pressure losses (Pa).

	Case 7 (20 m/s)		Case 14 (19 m/s)		
	Δ*P*_*S*1_	Δ*P*_*S*2_	Δ*P*_*S*1_	Δ*P*_*S*2_	Δ*P*_*S*3_
Measurement	256	260	486	528	410
LES simulation	258	254	491	539	417
